# Signatures of B Cell Receptor Repertoire Following *Pneumocystis* Infection

**DOI:** 10.3389/fmicb.2021.636250

**Published:** 2021-05-31

**Authors:** Han Sun, Hu-Qin Yang, Kan Zhai, Zhao-Hui Tong

**Affiliations:** Department of Respiratory and Critical Care Medicine, Beijing Institute of Respiratory Medicine, Beijing Chao-Yang Hospital, Capital Medical University, Beijing, China

**Keywords:** *Pneumocystis* infection, B cell receptor, single-cell BCR sequencing, single-cell RNA sequencing, somatic hypermutation

## Abstract

B cells play vital roles in host defense against *Pneumocystis* infection. However, the features of the B cell receptor (BCR) repertoire in disease progression remain unclear. Here, we integrated single-cell RNA sequencing and single-cell BCR sequencing of immune cells from mouse lungs in an uninfected state and 1–4 weeks post-infection in order to illustrate the dynamic nature of B cell responses during *Pneumocystis* infection. We identified continuously increased plasma cells and an elevated ratio of (IgA + IgG) to (IgD + IgM) after infection. Moreover, *Pneumocystis* infection was associated with an increasing naïve B subset characterized by elevated expression of the transcription factor *ATF3*. The proportion of clonal expanded cells progressively increased, while BCR diversity decreased. Plasma cells exhibited higher levels of somatic hypermutation than naïve B cells. Biased usage of V(D)J genes was observed, and the usage frequency of *IGHV9-3* rose. Overall, these results present a detailed atlas of B cell transcriptional changes and BCR repertoire features in the context of *Pneumocystis* infection, which provides valuable information for finding diagnostic biomarkers and developing potential immunotherapeutic targets.

## Introduction

*Pneumocystis jirovecii* pneumonia (PJP) is a severe opportunistic infectious disease found in immunocompromised patients (Schmidt et al., 2018). The incidence of PJP has been increasing in patients without human immunodeficiency virus (HIV) infection due to the widespread use of immunosuppressive medications (Bienvenu et al., 2016). Compared to HIV cases, the symptoms observed in HIV-negative PJP patients are more severe and abrupt, and the clinical outcome is worse (Guo et al., 2014), which indicates the significance of understanding the cellular and molecular basis of disease progression and developing better treatment strategies.

B cells have been demonstrated to play a vital role in host defense against *Pneumocystis* infection by antigen presentation and antibody production (Kolls, 2017; Otieno-Odhiambo et al., 2019). The B cell receptor (BCR), a B cell surface membrane immunoglobulin, possesses the ability to specifically recognize and bind antigens, with the complementarity-determining region 3 (CDR3) of the heavy chain as the major determinant of antibody specificity (Xu and Davis, 2000). The diversity of BCR repertoires in several infectious diseases has been explored, such as in coronavirus disease 2019 (Wen et al., 2020), dengue (Parameswaran et al., 2013), and chronic hepatitis C virus infection (Tucci et al., 2018). These results show that encountering a specific antigen could elicit clonal B-cell proliferation, thus altering the selective distribution of the BCR spectrum. *Pneumocystis*-specific BCR was reported to be required for adequate priming of T cells against *Pneumocystis*, rather than the mere presence of B cells (Opata et al., 2015), indicating the indispensable role of antigen-specific BCR in *Pneumocystis* infection. However, the specific characteristics of the BCR repertoire after *Pneumocystis* infection remains unknown.

The combination of single-cell RNA sequencing (scRNA-seq) and single-cell BCR sequencing enables us to simultaneously investigate the heterogeneity of the transcriptome and analyze the BCR features of B cell clones, which may lead to new insights into the host immune response (Zheng et al., 2017; Goldstein et al., 2019). Here, we integrated the transcriptome data and single-cell paired BCR analysis of B cells from mice lungs at different timepoints during *Pneumocystis* infection, aiming to elucidate the *Pneumocystis*-specific shuffling of the BCR repertoire and phenotypic characteristics of B cell clones.

## Materials and Methods

### Mice and *Pneumocystis* Lung Infection

C57BL/6 mice and severe combined immunodeficient mice (6–8 weeks of age) were purchased from Vital River Laboratory Animal Technology Co., Ltd. (Beijing, China). Animals were housed in specific pathogen-free conditions. *Pneumocystis murina* (ATCC, Manassas, VA, United States) was maintained in severe combined immunodeficient mice as previously described (Hu et al., 2017). Mouse models of *Pneumocystis* infection were prepared by intratracheally inoculating with 1 × 10^6^ cysts diluted in 100 μl phosphate-buffered saline under anesthesia, while non-infected mice were inoculated with 100 μl phosphate-buffered saline (Rong et al., 2019a). The animal studies were approved by the Capital Medical University Animal Care and Use Committee.

### Preparation of Single-Cell Suspensions and Cell Sorting

At specific time points, mice were sacrificed by exsanguination under deep anesthesia. Lungs were cut into pieces and transferred to a digestion medium containing DNAse I (Sigma), collagenase IV (Solarbio), and 10% fetal bovine serum in RPMI 1640 medium. After incubation for 20 min at 37°C with manual shaking every 5 min, samples were filtered with 40-μm nylon mesh (Biologix) and centrifuged at 400 × g for 6 min. Red blood cells were removed by lysing buffer (BD). Following surface-staining with Ghost Dye Red 780 (TONBO) and anti-mouse CD45 PerCP-Cyanine5.5 (BD) according to the operations manual, single-cell suspensions of the lung tissue from three mice were mixed together. Afterward, cell sorting was performed on a FACS Aria II (BD Biosciences) and CD45^+^ living cells were collected.

### scRNA-Seq and Preprocessing

Using Single Cell 5′ Library and Gel Bead kit (10x Genomics) and Chromium Single Cell A Chip kit (10x Genomics), cell suspensions were loaded onto a Chromium single cell controller (10x Genomics) to generate single-cell gel beads in the emulsion. The RNA of captured cells was released and barcoded through reverse transcription, and then the complementary DNA was amplified to establish the 5′ gene expression libraries. An Agilent 4200 system was used for quality assessment and after that, the libraries were sequenced using an Illumina Novaseq sequencer. Raw gene expression matrices were generated by Cell Ranger count pipeline with default parameters and mouse GRCm38/mm10 as the reference genome. The dataset integration of all five samples was achieved by Cell Ranger AGGR.

### Single-Cell Transcriptome Data Analysis

R software (v.4.0.2) with the Seurat (Butler et al., 2018) package (v.3.2.2) was applied for quality control. Low-quality cells were discarded if they met the following criteria: (1) the number of genes expressed were <200 or more than 4,000, or (2) > 25% unique molecular identifiers were derived from the mitochondrial genome. After normalizing the gene expression matrices by log normalization, the top 2,000 highly variable genes were calculated via the FindVariableFeatures function. The RunPCA function was conducted for linear dimensionality reduction, and non-linear dimensional reduction was performed with the RunUMAP function. Finally, cells were clustered together according to common features. The corresponding cell types of cell clusters were annotated based on the expressions of canonical markers.

### Differential Gene Expression Analysis and Functional Enrichment

Differential gene expression analysis was performed using the FindMarkers function in Seurat by a two-sided Wilcoxon test with Bonferroni correction. Genes with a maximum adjusted *p*-value of 0.01 and an absolute value of log_2_(fold change) > 0.5 were considered to be differentially expressed genes (DEGs) (Zhang J.Y. et al., 2020). Gene Ontology and Kyoto Encyclopedia of Genes and Genomes pathway analyses of DEGs were conducted using the Metascape (Zhou et al., 2019) webtool^1^.

### Single Cell Trajectory Analysis

We used the Monocle 2 (Qiu et al., 2017) package (version 2.16.0) to conduct pseudotime analysis. Ordering genes were calculated by the “differentialGeneTest” function (*q* < 0.01). To generate a two-dimensional projection of the underlying developmental trajectory, the “DDRTree” reduction method was used with default parameters. The “orderCells” function was applied to obtain cell ordering (pseudotime) along the lineage trajectory and the visualization function “plot_cell_trajectory” was used to plot the minimum spanning tree.

### Single-Cell BCR V(D)J Sequencing and Analysis

Full-length BCR V(D)J segments were enriched from amplified cDNA from 5′ libraries using a Chromium Single-Cell V(D)J Enrichment kit according to the manufacturer’s protocol. BCR sequences for each single B cell were assembled by Cell Ranger vdj pipeline (v.3.0.2). Only cells with both productive immunoglobulin heavy chains (IGH) and productive immunoglobulin light chains kappa (IGK) or lambda (IGL) were kept. If more than one heavy chain or light chain were detected in one cell, the chain with the highest amount of unique molecular identifiers was retained (Zheng et al., 2017).

A clonotype was defined as a unique combination of an IGH-IGK/IGL pair. A cell was considered to be clonally expanded if its clonotype was shared by at least two cells. The clonality of a clonotype could be indicated by the number of cells with the same clonotype. Using barcode information, B cells with prevalent BCR clonotypes were projected on a uniform manifold approximation and projection (UMAP) plot. The somatic hypermutation (SHM) rate of each B cell was defined as the percentage of mismatching nucleotides in the VH portion compared with the most similar germline gene (Kuri-Cervantes et al., 2020). Lineage analysis was performed on the protein sequences of the CDR3 region in both heavy and light chains. BCR sequences with the same V(D)J assignment were grouped together into a lineage if their CDR3 sequences differed by no more than one amino acid (Jiang et al., 2013). Plots of the lineage structures were generated with Cytoscape (v.3.5.1).

## Results

### Study Design and Single-Cell Profiling of B Cells

To assess the signatures of B-cell receptor diversity in B lymphocytes following *Pneumocystis* infection, we performed scRNA-seq and single-cell BCR sequencing on CD45^+^ immune cells from the lung tissue of uninfected mice (0 w) and mouse models at 1–4 weeks post-infection (1 w, 2 w, 3 w, and 4 w). Each sample contained CD45^+^ cells from three mice. Based on the single-cell transcriptome data, a total of 11,062 B cells were obtained after filtering. We integrated the scRNA-seq and single-cell paired BCR analysis for each subject, and only cells with full-length productive paired IGH-IGK/IGL chains were retained. Finally, 3,280 B lymphocytes were included for subsequent analysis.

### Features of B Cell Subsets After *Pneumocystis* Infection

**FIGURE 1:**
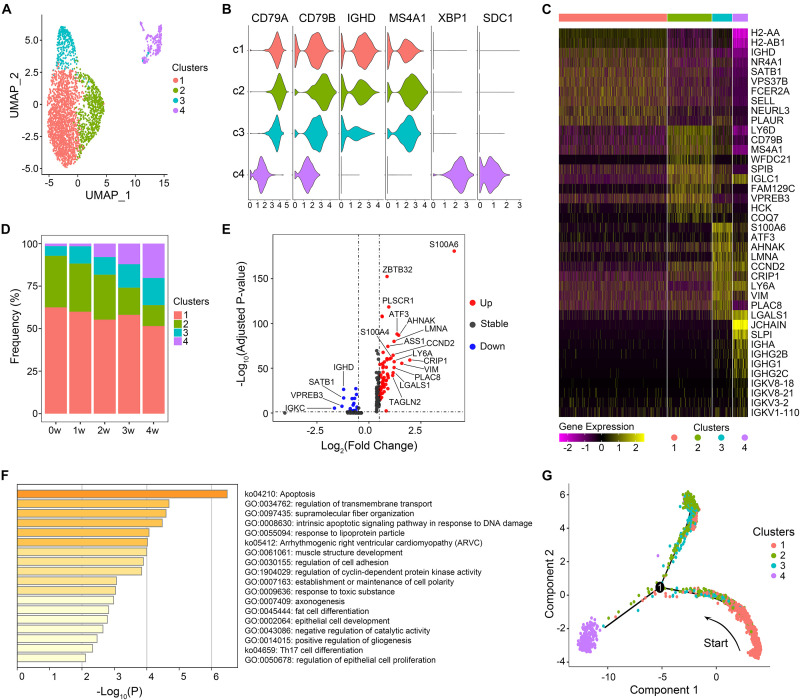
Features of B cell subsets. **(A)** Uniform manifold approximation and projection (UMAP) projection of B cells from all five samples. Each dot corresponds to a single cell, colored according to cell clusters. **(B)** Violin plots show expression distribution of canonical cell markers in B cell subsets. **(C)** The expression of the top 10 marker genes of each cluster. **(D)** Proportion of B cell clusters in each sample. **(E)** Differentially expressed genes of cluster 3 in comparison with all other cells. Genes with a maximum adjusted *p*-value of 0.01 and an absolute value of log_2_(fold change) > 0.5 were considered to be differentially expressed genes. **(F)** Gene enrichment analyses of upregulated differentially expressed genes of cluster 3. **(G)** Trajectory reconstruction of all B cells through pseudotime analysis.

UMAP clustering analyses were performed on B lymphocytes, yielding four cell clusters (Figure 1A). According to the differential expression of canonical genes, B cells were categorized into naïve B (clusters 1, 2, and 3) and plasma cells (cluster 4). Naïve B cells expressed high levels of *IGHD* and *MS4A1*, while plasma cells were identified on the basis of high expression of *XBP1* and *SDC1* (Figure 1B).

We then explored the gene expression pattern of each B cell subset (Figure 1C) and the relative frequencies of the four clusters among each sample (Figure 1D). Cluster 1 accounted for the largest portion of B cells in each sample. *IGHD* and homing marker *SELL* were present at high levels in cluster 1, suggesting that cluster 1 was retained in a relatively naïve state. Genes related to B cell activation (*CD79B*, *MS4A1*, and *CD24A*) were markedly expressed in cluster 2 (Figure 1C and Supplementary Figure 1A). DEGs preferentially expressed in cluster 2 were mainly enriched in B cell activation and B cell differentiation (Supplementary Figure 1B), indicating that cluster 2 was in a relatively activated state. The proportion of cluster 3 in total B cells presented an increasing trend over time, from 5.7% in the uninfected sample to 16.0% in the 4 w sample. Activating transcription factor-3 (ATF3), a key regulator of inflammatory responses, was significantly upregulated in cluster 3 (Figure 1E). In addition, the high level of *TAGLN2* expression, which was reported to be a potential marker of activated B cells (Kiso et al., 2017), implied that cluster 3 was also activated to some extent. DEGs significantly enriched in cluster 3 were associated with regulation of transmembrane transport, cell adhesion, and Th17 cell differentiation (Figure 1F), indicating the effector functions of cluster 3. Compared with that in the uninfected mice, the percentage of plasma cells rose continuously during the infection process, suggesting a key role for plasma cells in the control of *Pneumocystis* infection and the development of adaptive immunity by inducing specific antibodies.

In order to discover the cell-state transitions, we performed a pseudotime analysis using Monocle 2, an algorithm for the lineage reconstruction of biological processes based on transcriptional similarity (Figure 1G). A trajectory of B cell populations was ordered from a starting point of cluster 1 and bifurcated into two diverse branches, with clusters 2 and 3 in one terminal, while plasma cells were in the other terminal, representing two major cell lineages in the process of B cell differentiation after *Pneumocystis* infection. The cell trajectory analysis supported the above speculation that cluster 1 is in a more primitive stage, which could transit to relatively activated naïve B subsets (clusters 2 and 3) and plasma cells (cluster 4) in response to *Pneumocystis* antigenic stimulation.

### Clonal Expansion and IgH Class Switching of B Cells After *Pneumocystis* Infection

To quantitatively assess the clonal diversity of B cells, the D50 value (Kuo et al., 2019) was calculated. The D50 value of the 0 w sample was 49.9, which was higher than the infected samples (49.8, 48.3, 48.0, and 47.3 for the 1 w, 2 w, 3 w, and 4 w samples, respectively). The D50 value analysis showed that B-cell diversity decreased after *Pneumocystis* infection.

**FIGURE 2:**
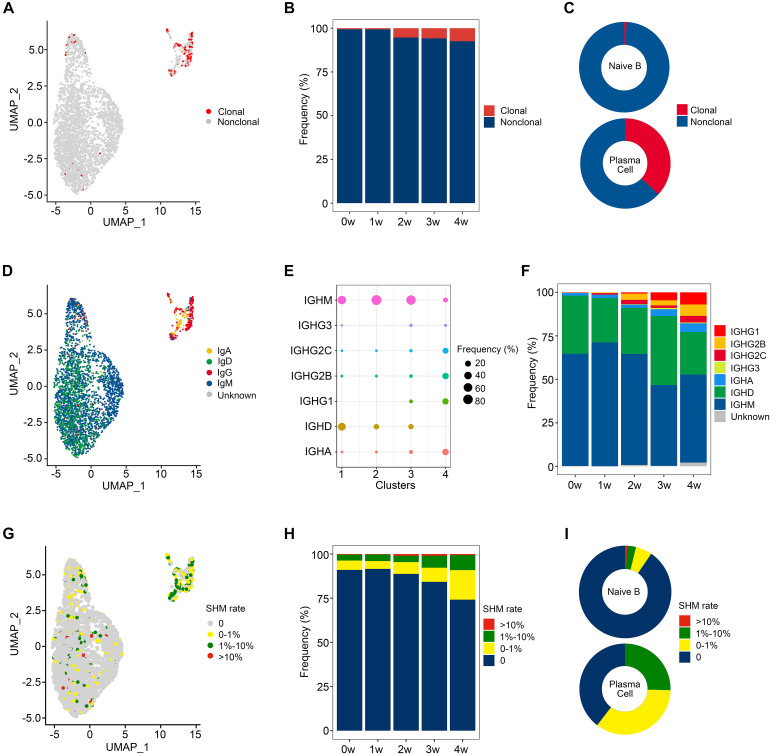
Clonal expansion and immunoglobulin class switching of B cells after *Pneumocystis* infection. **(A)** Uniform manifold approximation and projection (UMAP) plot shows the distribution of clonal expanded B cells. **(B)** The proportion of clonal expanded B cells in each sample. **(C)** The pie plot shows the proportion of clonal expanded B cells in naïve B and plasma cells. **(D)** UMAP plot of B cells colored by immunoglobulin heavy chain expression. **(E)** The proportion of each immunoglobulin isotype in B cell subsets. **(F)** The proportion of different immunoglobulin isotype in each sample. **(G)** UMAP plot of B cells colored by somatic hypermutation (SHM) rate. **(H)** The proportion of B cells with SHM in each sample. **(I)** The pie plot shows the proportion of B cells with SHM in naïve B and plasma cells.

We then evaluated the distribution of clonal expanded B cells. As seen in the UMAP projections, clonal expanded B cells mainly belonged to plasma cells (79.2%), while naïve B cells exhibited little clonality (Figure 2A). The percentage of clonal expanded cells was 4.6% in ATF3^+^ naïve B cells (cluster 3), which is higher than those in the other 2 subclusters of naïve B cells, both 0.4%. According to the bar plot (Figure 2B), the proportion of clonal cells progressively increased from 0.6% (0 w) to 7.5% (4 w) after *Pneumocystis* infection. Among the B cell clusters, 93.1% of naïve B cells owned a unique BCR clonotype, while 36.9% of plasma cells showed clonal expansions (Figure 2C).

IgH class switching occurs after responding to antigens and confers distinct functional characteristics to B cells (Stavnezer and Schrader, 2014). The delineation of isotypes is therefore essential for the comprehensive analysis of the BCR repertoire. To further investigate the dynamic changes of IgH class switching after *Pneumocystis* infection, we evaluated the distribution of IgA, IgD, IgG, and IgM, as IgE was not detected (Figure 2D). A total of 64.7% of naïve B cells expressed the IgM isotype, followed by IgD (32.2%), IgG (1.6%), and IgA (0.7%). Plasma cells preferentially expressed the IgG isotype (64.9%), and IgA made up a relatively small proportion (25.4%; Figure 2E). IgM remained the predominant immunoglobulin, whereas the abundance of IgG apparently increased with the disease progression (Figure 2F). Compared to the uninfected state, the ratio of (IgA + IgG) to (IgD + IgM) increased after infection, suggesting that *Pneumocystis* induced an intensive antibody response.

SHM in the immunoglobulin variable gene allows for the generation of high-affinity antibodies (Wrammert et al., 2008). We assessed the SHM of VH portion in every unique sequence. The mutation rate was divided into four groups (0, 0–1%, 1–10%, and > 10%), which are shown in the UMAP plot (Figure 2G). Less than 10% of B cells exhibited SHM in sample 0 w and sample 1 w, while an increasing proportion of cells experienced SHM in the 2 w, 3 w, and 4 w samples (11.2%, 15.7%, and 25.8%, respectively, Figure 2H). A total of 90.5% of naïve B cells were unmutated, while 60.4% of plasma cells experienced SHM (Figure 2I).

### CDR3 Length and Specific Rearrangements of V(D)J Genes After *Pneumocystis* Infection

**FIGURE 3:**
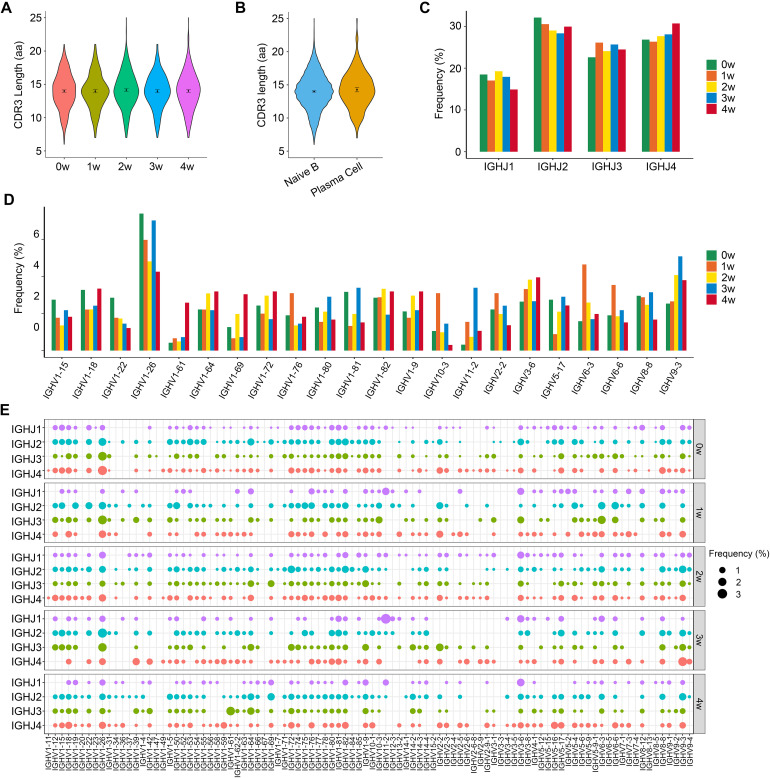
The distribution of immunoglobin heavy chains (IGH) CDR3 length and selective usage of V and J genes. **(A)** Violin plots show the distribution of IGH CDR3 amino acid length in each sample. **(B)** Distribution of IGH CDR3 amino acid length in naïve B and plasma cells. **(C)** Selective usage of J gene segments in each sample. **(D)** Usage of some V gene segments in each sample. **(E)** Bubble chart shows the usage frequency of V–J gene combination in each sample.

We then calculated the CDR3 length of the BCR heavy chain, since the length of the CDR3 region affects the three-dimensional structure of the CDR3 ring, thus influencing antigen-binding specificity. The length of CDR3 in total B cells ranged from 6 to 25 amino acids (aa), with an average of 14 aa for each sample (Figure 3A). There was no significant difference in the CDR3 length between naïve B and plasma cells, and their average CDR3 aa lengths were both 14 aa. For naïve B cells, the length of CDR3 varied from 6 to 25 aa, and for plasma cells, the CDR3 length varied from 7 to 25 aa (Figure 3B).

To study biased V(D)J rearrangements of the BCR heavy chain, we determined differences in the usage frequency of the V, D, and J gene segments across all five samples. According to the sequencing results, a total of 96 IGHV gene segments, 13 IGHD gene segments, and 4 IGHJ gene segments were identified in the whole B cells.

We generated a distribution histogram of IGHJ genes usage frequency for total B cells and discovered that *IGHJ2* was most frequently used in the first four samples, while the usage of *IGHJ4* gradually increased from sample 1 w to sample 4 w, and became the most frequently used IGHJ gene segment in the 4 w sample. *IGHJ1* shared the lowest utilization (Figure 3C).

The selective usage of IGHV genes was analyzed (Figure 3D and Supplementary Figures 2A,B). For total B cells, we detected an over-representation of the *IGHV1* family, especially *IGHV1-26*, which occupied the most frequent IGHV gene segment in each sample, with frequencies of 7.4%, 6.0%, 4.8%, 7.1%, and 4.3% for the 0 w, 1 w, 2 w, 3 w, and 4 w samples, respectively. It is remarkable that *IGHV9-3* was gradually enriched following the infectious process and peaked at 3 w post-infection (5.1%). In addition, we observed the over-representation of *IGHV6-3* in the 1 w sample (4.6%) and *IGHV 1-61* in the 4 w sample (2.6%).

A total of 332 unique V–J combinations were identified in the whole B cells (Figure 3E). The top paired V–J frequency in the uninfected sample was *IGHV1-26*/*IGHJ4* (2.6%), while those in the 1 w, 2 w, 3 w, and 4 w samples were *IGHV1-26*/*IGHJ3* (2.4%), *IGHV1-72*/*IGHJ2* (1.6%), *IGHV11-2*/*IGHJ1* (3.1%), and *IGHV1-61*/*IGHJ3* (2.3%), respectively. A total of 1,386 IGHV-IGHD-IGHJ rearrangements were found in all B cells. The most preferred rearrangements in the 0 w, 1 w, 2 w, 3 w, and 4 w samples were *IGHV1-26*/*IGHD1-1*/*IGHJ2* (0.7%), *IGHV1-76*/*IGHD1-1*/*IGHJ2* (1.0%), *IGHV3-6*/*IGHD1-1*/*IGHJ1* (1.1%), *IGHV11-2*/*IGHD2-6*/*IGHJ1* (2.7%), and *IGHV1-61*/*IGHD1-1*/*IGHJ3* (2.5%), respectively.

**FIGURE 4:**
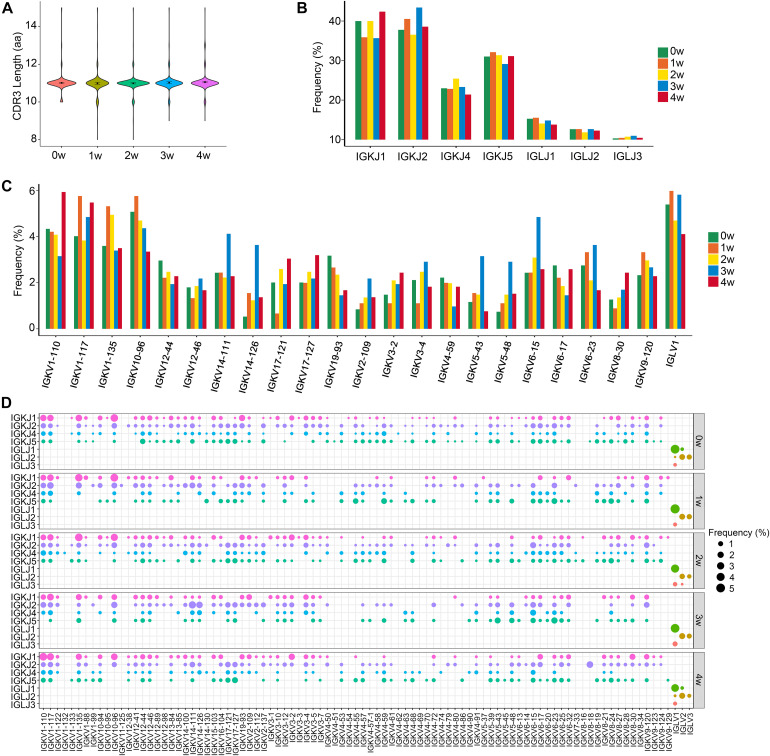
The distribution of immunoglobin light chains kappa (IGK)/immunoglobin light chains lambda (IGL) CDR3 length and selective usage of V and J genes. **(A)** Distribution of IGK/IGL CDR3 amino acid length in total B cells. **(B)** Selective usage of J gene segments in total B cells. **(C)** Selective usage of some IGK/L V gene segments in each sample. **(D)** Bubble chart shows the usage frequency of V–J gene combination in each sample.

We also compared the CDR3 length and the usage of V and J genes in BCR light chains among each sample. Light chain analysis demonstrated preferential IGK use with an IGK/IGL ratio of 12.2:1. The average CDR3 lengths of BCR light chains were 11 aa in all five samples (Figure 4A). The usage pattern of J gene segments was displayed (Figure 4B), and shuffling of the V gene usage preference and V–J combinations in light chains was observed (Figures 4C,D and Supplementary Figures 3A,B).

In order to explore *Pneumocystis-*specific BCRs, we performed lineage analysis on all B cells. Two sequences were clustered into a lineage if they shared the same V(D)J combination and their CDR3 region differed by no more than one amino acid. The lineage structures of each sample are shown in Supplementary Figures 4–8. Lineages which contained only one cell remained dominant in all five samples and few sequences from different samples could be clustered into the same lineage. A total of 11.3% of plasma cells in the 4 w sample were grouped together into a lineage, with a SHM rate of 0–1.4%. These IgG1-expressing B cells shared the IGH-IGK pair of *IGHV1-61*/*IGHD1-1*/*IGHJ3*–*IGKV8-18*/*IGKJ2*. The protein sequences of the CDR3 region in the heavy chain and light chain were ARNGYYGSSRFAYW and CQHNHGS(T)FLPYTF, respectively.

## Discussion

PJP is a fatal disease in the non-HIV infected immunosuppressed population. Host immune response against *Pneumocystis* infection not only plays an antifungal role, but it also leads to simultaneous pathogenic injury of the tissue, which determines the disease severity, progression, and outcome. B cells represent a major component of the humoral immune system. In this study, we explored the B cell transcriptome profiles and BCR repertoires in mouse lungs before and after *Pneumocystis* infection to investigate the immune mechanisms underlying the responsiveness to the pathogen. Our integrated single-cell transcriptomic and BCR sequencing analysis resulted in several insights into the immunobiology of PJP.

In our study, *Pneumocystis* infection caused the elevated proportion of a subcluster of naïve B cells, with the high expression of the *ATF3* gene. ATF3 is a fundamental transcription factor in the endoplasmic reticulum-oxidative stress pathway, and it has a significant function in the cellular adaptive-response network. In mouse models, ATF3 could be superinduced by reactive oxygen species and protect against endotoxic shock by inhibiting innate cytokines (Hoetzenecker et al., 2011). In addition, ATF3 facilitates the pathogen clearance in *S. pneumoniae* infection by promoting IL-17A production in γδ T cells (Lee et al., 2018), and it inhibits the secretion of inflammatory cytokines induced by *Mycoplasma pneumonia in vitro* and *in vivo* (Wang et al., 2017). As evidenced by our analysis, the ATF3^+^ B cells highly expressed genes associated with regulation of transmembrane transport, cell adhesion, and Th17 cell differentiation. The immunologic function of ATF3 in PJP remains in need of further exploration.

B cell responses to antigens are characterized by the activation of reactive B cell clones. Based on our results, the percentage of clonal expanded B cells continuously increased after *Pneumocystis* infection, while the BCR diversity exhibited a downward tendency. The BCR is an essential functional receptor, allowing B cells to specifically recognize antigens. Once the antigen is recognized, B cells proliferate and undergo affinity maturation by accumulation of somatic mutations (Roy et al., 2017). The clonal expansion of B cells was caused by chronic stimulation of foreign antigens, and the reduction of BCR diversity reflected the selection of B cells that were adapted to recognize *Pneumocystis*-specific antigens. Over 60% of plasma cells exhibited SHM, suggesting enhanced BCR affinity and specificity.

As the most variable region of the BCR sequence, the CDR3 region directly determines the specificity of antigen binding to the BCR (Bashford-Rogers et al., 2018). The length of CDR3 was reported to be associated with antibody polyreactivity and autoimmunity (Meffre et al., 2001). The average CDR3 length in patients with systemic lupus erythematosus or systemic sclerosis was significantly shorter than that in the healthy controls (Odendahl et al., 2000; Shi et al., 2020), and B cell clones from patients with rheumatoid arthritis were enriched for longer heavy chain CDR3 sequences (Doorenspleet et al., 2014). Moreover, longer CDR3 length was demonstrated to be preferentially selected during persistent infection (Breden et al., 2011). In this study, we measured the CDR3 aa lengths of BCR heavy chains and their distribution among each sample. The average CDR3 aa lengths in the five samples were all 14 aa. Although a higher percentage of cells with CDR3 aa lengths longer than 20 aa was detected in plasma cells, there was no significant difference in the CDR3 aa lengths between naïve B and plasma cells.

The differential use of IGHV genes contributes to the diversity of the BCR repertoire. Compared with the uninfected control sample, significant changes in IGHV gene segment usage were observed in samples with *Pneumocystis* infection. In addition to *IGHV1-26*, which remained the most frequently used IGHV gene segment in each sample, the usage frequency of *IGHV9-3* elevated after *Pneumocystis* infection, and it became the second most frequently used IGHV gene segment in the 2 and 3 w samples. In terms of the previous research of our group, the fungal burden of *Pneumocystis* peaked 3 weeks post-infection and then gradually cleared (Li et al., 2018; Rong et al., 2019b; Zhang C. et al., 2020). The increased usage of *IGHV9-3* might provide a direction for further study of finding prognostic or diagnostic biomarkers for *Pneumocystis* pneumonia.

The single-cell resolution data acquired from scRNA-seq and single-cell BCR V(D)J sequencing enabled us to carry out lineage analysis based on the similarity of protein sequences in CDR3 regions. Due to the relative shallow sampling of immune cells from each mouse, most of the BCR lineages did not show any enrichment, and contained only a single cell. A lineage of plasma cells expressed the protein sequence of ARNGYYGSSRFAYW and CQHNHGS(T)FLPYTF in the heavy chain and light chain CDR3 region, constituting 11.3% of plasma cells in the 4 w sample. However, no naïve B cells in the 4 w sample expressed this sequence and no BCRs from other samples could be clustered into the same lineage. It remains to be confirmed that whether this CDR3 sequence was induced by antigen stimulation, and its affinity with *Pneumocystis*-specific antigens needs to be detected.

Taken together, our work integrated the transcriptomic data and single-cell paired BCR profiles, revealing the dynamic change of the BCR repertoire during *Pneumocystis* infection. This study identified the alterations in B cell subtypes and BCR clonal expansion, laying the foundation for further understanding of host immune mechanisms against *Pneumocystis* infection. It is likely to provide novel insight into finding diagnostic biomarkers and developing effective immunotherapies for PJP.

## Data Availability Statement

The datasets presented in this study can be found in onlinerepositories. The names of the repository/repositoriesand accession number(s) can be found below: https://www.ncbi.nlm.nih.gov/ and GSE157627; https://www.ncbi.nlm.nih.gov/, GSE162533.

## Ethics Statement

The animal study was reviewed and approved by the Capital Medical University Animal Care and Use Committee.

## Author Contributions

Z-HT and KZ: conception and design. HS and H-QY: collection and assembly of data. HS: data analysis and interpretation. All authors: manuscript writing, final approval of manuscript.

## Conflict of Interest

The authors declare that the research was conducted in the absence of any commercial or financial relationships that could be construed as a potential conflict of interest.

## Footnotes

1 www.metascape.org

## Supplementary Material

Supplementary Figure 1 Gene enrichment analyses of differentially expressed genes in cluster 2. **(A)** Differentially expressed genes of cluster 2 in comparison with all other cells. Genes with a maximum adjusted *p*-value of 0.01 and an absolute value of log_2_(fold change) > 0.5 were considered to be differentially expressed genes. **(B)** Gene enrichment analyses of upregulated differentially expressed genes of cluster 2.

Supplementary Figure 2 Selective usage of immunoglobin heavy chains (IGH) V genes. **(A)** Selective usage of V gene segments in each sample. **(B)** Heatmaps of V gene segment usage.

Supplementary Figure 3 Selective usage of immunoglobin light chains kappa/lambda (IGK/L) V genes. **(A)** Selective usage of V gene segments in each sample. **(B)** Heatmaps of V gene segment usage.

Supplementary Figure 4 Lineage analysis of all B cells in sample 0 w. Each dot represents a unique CDR3 protein sequence. The area of a dot is proportional to the number of cells with identical CDR3 protein sequences. Two dots are linked if they differ by one amino acid in the CDR3 region.

Supplementary Figure 5 Lineage analysis of all B cells in sample 1 w. Each dot represents a unique CDR3 protein sequence. The area of a dot is proportional to the number of cells with identical CDR3 protein sequences. Two dots are linked if they differ by one amino acid in the CDR3 region.

Supplementary Figure 6 Lineage analysis of all B cells in sample 2 w. Each dot represents a unique CDR3 protein sequence. The area of a dot is proportional to the number of cells with identical CDR3 protein sequences. Two dots are linked if they differ by one amino acid in the CDR3 region.

Supplementary Figure 7 Lineage analysis of all B cells in sample 3 w. Each dot represents a unique CDR3 protein sequence. The area of a dot is proportional to the number of cells with identical CDR3 protein sequences. Two dots are linked if they differ by one amino acid in the CDR3 region.

Supplementary Figure 8 Lineage analysis of all B cells in sample 4 w. Each dot represents a unique CDR3 protein sequence. The area of a dot is proportional to the number of cells with identical CDR3 protein sequences. Two dots are linked if they differ by one amino acid in the CDR3 region. The lineage with a red border showed enrichment, with CDR3 protein sequences in the heavy chain and light chain of ARNGYYGSSRFAYW and CQHNHGS(T)FLPYTF, respectively.

## Abbreviations

ATF3: activating transcription factor-3
BCR: B cell receptor
CDR3: complementarity-determining region 3
DEGs: differentially expressed genes
HIV: human immunodeficiency virus
IGH: immunoglobulin heavy chains
IGK: immunoglobulin light chains kappa
IGL: immunoglobulin light chains lambda
PJP: *Pneumocystis jirovecii* pneumonia
SHM: somatic hypermutation
scRNA-seq: single-cell RNA sequencing
UMAP: uniform manifold approximation and projection.

## References

[B1] Bashford-Rogers R. J. M. Smith K. G. C. Thomas D. C. (2018). Antibody repertoire analysis in polygenic autoimmune diseases. *Immunology* 155 3–17. 10.1111/imm.12927 29574826 PMC6099162

[B2] Bienvenu A. L. Traore K. Plekhanova I. Bouchrik M. Bossard C. Picot S. (2016). Pneumocystis pneumonia suspected cases in 604 non-HIV and HIV patients. *Int. J. Infect Dis.* 46 11–17. 10.1016/j.ijid.2016.03.018 27021532

[B3] Breden F. Lepik C. Longo N. S. Montero M. Lipsky P. E. Scott J. K. (2011). Comparison of antibody repertoires produced by HIV-1 infection, other chronic and acute infections, and systemic autoimmune disease. *PLoS One* 6:e16857. 10.1371/journal.pone.0016857 21479208 PMC3068138

[B4] Butler A. Hoffman P. Smibert P. Papalexi E. Satija R. (2018). Integrating single-cell transcriptomic data across different conditions, technologies, and species. *Nat. Biotechnol.* 36 411–420. 10.1038/nbt.4096 29608179 PMC6700744

[B5] Doorenspleet M. E. Klarenbeek P. L. de Hair M. J. van Schaik B. D. Esveldt R. E. van Kampen A. H. (2014). Rheumatoid arthritis synovial tissue harbours dominant B-cell and plasma-cell clones associated with autoreactivity. *Ann. Rheum. Dis.* 73 756–762. 10.1136/annrheumdis-2012-202861 23606709

[B6] Goldstein L. D. Chen Y. J. Wu J. Chaudhuri S. Hsiao Y. C. Schneider K. (2019). Massively parallel single-cell B-cell receptor sequencing enables rapid discovery of diverse antigen-reactive antibodies. *Commun. Biol.* 2:304. 10.1038/s42003-019-0551-y 31428692 PMC6689056

[B7] Guo F. Chen Y. Yang S. L. Xia H. Li X. W. Tong Z. H. (2014). Pneumocystis pneumonia in HIV-infected and immunocompromised non-HIV infected patients: a retrospective study of two centers in China. *PLoS One* 9:e101943. 10.1371/journal.pone.0101943 25029342 PMC4100803

[B8] Hoetzenecker W. Echtenacher B. Guenova E. Hoetzenecker K. Woelbing F. Bruck J. (2011). ROS-induced ATF3 causes susceptibility to secondary infections during sepsis-associated immunosuppression. *Nat. Med.* 18 128–134. 10.1038/nm.2557 22179317 PMC3555699

[B9] Hu Y. Wang D. Zhai K. Tong Z. (2017). Transcriptomic analysis reveals significant B lymphocyte suppression in corticosteroid-treated hosts with pneumocystis pneumonia. *Am. J. Respir. Cell Mol. Biol.* 56 322–331. 10.1165/rcmb.2015-0356OC 27788015

[B10] Jiang N. He J. Weinstein J. A. Penland L. Sasaki S. He X. S. (2013). Lineage structure of the human antibody repertoire in response to influenza vaccination. *Sci. Transl. Med.* 5:171ra119. 10.1126/scitranslmed.3004794 23390249 PMC3699344

[B11] Kiso K. Yoshifuji H. Oku T. Hikida M. Kitagori K. Hirayama Y. (2017). Transgelin-2 is upregulated on activated B-cells and expressed in hyperplastic follicles in lupus erythematosus patients. *PLoS One* 12:e0184738. 10.1371/journal.pone.0184738 28910360 PMC5599031

[B12] Kolls J. K. (2017). An emerging role of B cell immunity in susceptibility to pneumocystis pneumonia. *Am. J. Respir. Cell Mol. Biol.* 56 279–280. 10.1165/rcmb.2016-0360ED 28248133 PMC5359542

[B13] Kuo H. C. Pan C. T. Huang Y. H. Huang F. C. Lin Y. S. Li S. C. (2019). Global investigation of immune repertoire suggests kawasaki disease has infectious cause. *Circ. J.* 83 2070–2078. 10.1253/circj.CJ-19-0206 31378745

[B14] Kuri-Cervantes L. Pampena M. B. Meng W. Rosenfeld A. M. Ittner C. A. G. Weisman A. R. (2020). Comprehensive mapping of immune perturbations associated with severe COVID-19. *Sci. Immunol.* 5:eabd7114. 10.1126/sciimmunol.abd7114 32669287 PMC7402634

[B15] Lee S. Kim G. L. Kim N. Y. Kim S. J. Ghosh P. Rhee D. K. (2018). ATF3 stimulates IL-17A by regulating intracellular Ca(2+)/ROS-dependent IL-1beta activation during streptococcus pneumoniae infection. *Front. Immunol.* 9:1954. 10.3389/fimmu.2018.01954 30214444 PMC6125349

[B16] Li T. Rong H. M. Zhang C. Zhai K. Tong Z. H. (2018). IL-9 deficiency promotes pulmonary Th17 response in murine model of pneumocystis infection. *Front. Immunol.* 9:1118. 10.3389/fimmu.2018.01118 29887863 PMC5980981

[B17] Meffre E. Milili M. Blanco-Betancourt C. Antunes H. Nussenzweig M. C. Schiff C. (2001). Immunoglobulin heavy chain expression shapes the B cell receptor repertoire in human B cell development. *J. Clin. Invest.* 108 879–886. 10.1172/JCI13051 11560957 PMC200933

[B18] Odendahl M. Jacobi A. Hansen A. Feist E. Hiepe F. Burmester G. R. (2000). Disturbed peripheral B lymphocyte homeostasis in systemic lupus erythematosus. *J. Immunol.* 165 5970–5979. 10.4049/jimmunol.165.10.5970 11067960

[B19] Opata M. M. Hollifield M. L. Lund F. E. Randall T. D. Dunn R. Garvy B. A. (2015). B lymphocytes are required during the early priming of CD4+ T cells for clearance of pneumocystis infection in mice. *J. Immunol.* 195 611–620. 10.4049/jimmunol.1500112 26041535 PMC4491042

[B20] Otieno-Odhiambo P. Wasserman S. Hoving J. C. (2019). The contribution of host cells to pneumocystis immunity: an update. *Pathogens* 8:52. 10.3390/pathogens8020052 31010170 PMC6631015

[B21] Parameswaran P. Liu Y. Roskin K. M. Jackson K. K. Dixit V. P. Lee J. Y. (2013). Convergent antibody signatures in human dengue. *Cell Host Microbe* 13 691–700. 10.1016/j.chom.2013.05.008 23768493 PMC4136508

[B22] Qiu X. Mao Q. Tang Y. Wang L. Chawla R. Pliner H. A. (2017). Reversed graph embedding resolves complex single-cell trajectories. *Nat. Methods* 14 979–982. 10.1038/nmeth.4402 28825705 PMC5764547

[B23] Rong H. M. Li T. Zhang C. Wang D. Hu Y. Zhai K. (2019a). IL-10-producing B cells regulate Th1/Th17-cell immune responses in pneumocystis pneumonia. *Am. J. Physiol. Lung Cell Mol. Physiol.* 316 L291–L301. 10.1152/ajplung.00210.2018 30284926

[B24] Rong H. M. Qian X. J. Zhang C. Li T. Tong Z. H. (2019b). IL-17 inversely correlated with IL-10 via the STAT3 gene in pneumocystis-infected mice. *Mediators Inflamm.* 2019:6750861. 10.1155/2019/6750861 31582901 PMC6754930

[B25] Roy B. Neumann R. S. Snir O. Iversen R. Sandve G. K. Lundin K. E. A. (2017). High-throughput single-cell analysis of B cell receptor usage among autoantigen-specific plasma cells in celiac disease. *J. Immunol.* 199 782–791. 10.4049/jimmunol.1700169 28600290

[B26] Schmidt J. J. Lueck C. Ziesing S. Stoll M. Haller H. Gottlieb J. (2018). Clinical course, treatment and outcome of pneumocystis pneumonia in immunocompromised adults: a retrospective analysis over 17 years. *Crit. Care* 22:307. 10.1186/s13054-018-2221-8 30454031 PMC6245758

[B27] Shi X. Shao T. Huo F. Zheng C. Li W. Jiang Z. (2020). An analysis of abnormalities in the B cell receptor repertoire in patients with systemic sclerosis using high-throughput sequencing. *PeerJ* 8:e8370. 10.7717/peerj.8370 31988805 PMC6968515

[B28] Stavnezer J. Schrader C. E. (2014). IgH chain class switch recombination: mechanism and regulation. *J. Immunol.* 193 5370–5378. 10.4049/jimmunol.1401849 25411432 PMC4447316

[B29] Tucci F. A. Kitanovski S. Johansson P. Klein-Hitpass L. Kahraman A. Durig J. (2018). Biased IGH VDJ gene repertoire and clonal expansions in B cells of chronically hepatitis C virus-infected individuals. *Blood* 131 546–557. 10.1182/blood-2017-09-805762 29242186

[B30] Wang J. Cheng W. Wang Z. Xin L. Zhang W. (2017). ATF3 inhibits the inflammation induced by mycoplasma pneumonia in vitro and in vivo. *Pediatr. Pulmonol.* 52 1163–1170. 10.1002/ppul.23705 28440917

[B31] Wen W. Su W. Tang H. Le W. Zhang X. Zheng Y. (2020). Immune cell profiling of COVID-19 patients in the recovery stage by single-cell sequencing. *Cell Discov.* 6:31. 10.1038/s41421-020-0168-9 32377375 PMC7197635

[B32] Wrammert J. Smith K. Miller J. Langley W. A. Kokko K. Larsen C. (2008). Rapid cloning of high-affinity human monoclonal antibodies against influenza virus. *Nature* 453 667–671. 10.1038/nature06890 18449194 PMC2515609

[B33] Xu J. L. Davis M. M. (2000). Diversity in the CDR3 region of V(H) is sufficient for most antibody specificities. *Immunity* 13 37–45. 10.1016/s1074-7613(00)00006-6 10933393

[B34] Zhang C. Rong H. M. Li T. Zhai K. Tong Z. H. (2020). PD-1 deficiency promotes macrophage activation and T-helper cell type 1/T-helper cell type 17 response in pneumocystis pneumonia. *Am. J. Respir. Cell Mol. Biol.* 62 767–782. 10.1165/rcmb.2019-0234OC 32048861

[B35] Zhang J. Y. Wang X. M. Xing X. Xu Z. Zhang C. Song J. W. (2020). Single-cell landscape of immunological responses in patients with COVID-19. *Nat. Immunol.* 21 1107–1118. 10.1038/s41590-020-0762-x 32788748

[B36] Zheng C. Zheng L. Yoo J. K. Guo H. Zhang Y. Guo X. (2017). Landscape of infiltrating T cells in liver cancer revealed by single-cell sequencing. *Cell* 169 1342–1356.e16. 10.1016/j.cell.2017.05.035 28622514

[B37] Zhou Y. Zhou B. Pache L. Chang M. Khodabakhshi A. H. Tanaseichuk O. (2019). Metascape provides a biologist-oriented resource for the analysis of systems-level datasets. *Nat. Commun.* 10:1523. 10.1038/s41467-019-09234-6 30944313 PMC6447622

