# Supplementary figures and images for: Signatures of B Cell Receptor Repertoire Following Pneumocystis Infection
Source: Front Microbiol. 2021 May 31;12:636250. doi: 10.3389/fmicb.2021.636250 (PMC8202503; doi:10.3389/fmicb.2021.636250)

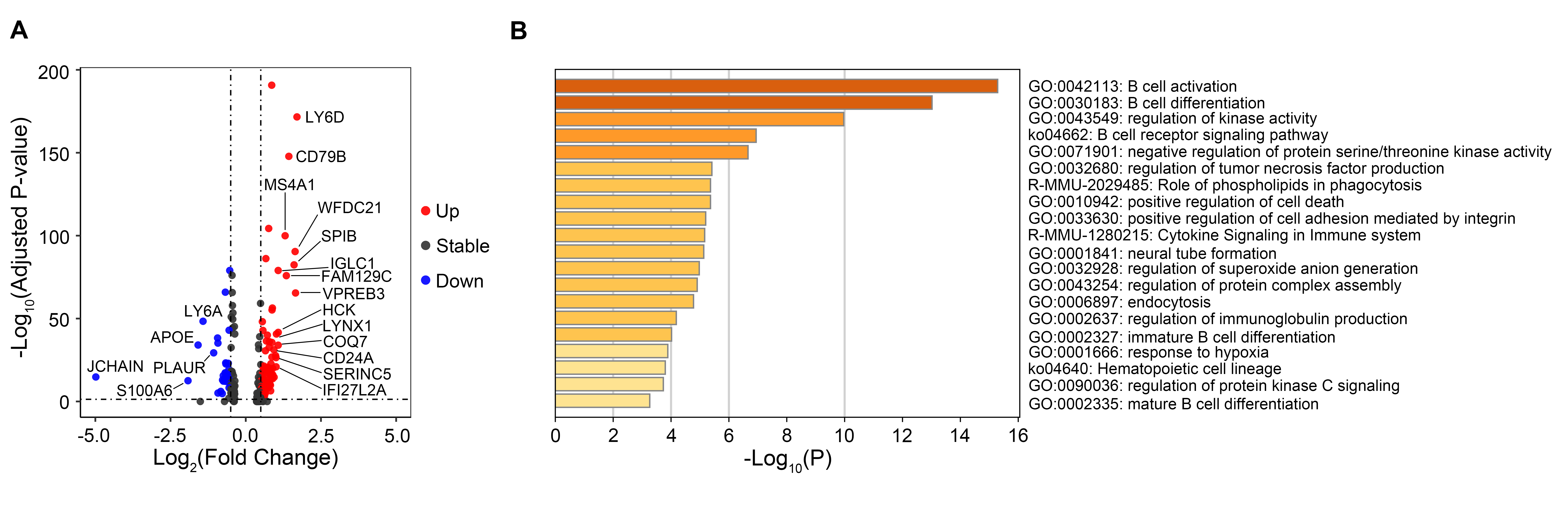

Supplement: Supplementary Figure 1 — Gene enrichment analyses of differentially expressed genes in cluster 2. (A) Differentially expressed genes of cluster 2 in comparison with all other cells. Genes with a maximum adjusted p-value of 0.01 and an absolute value of log2(fold change) > 0.5 were considered to be differentially expressed genes. (B) Gene enrichment analyses of upregulated differentially expressed genes of cluster 2. [file Image_1.TIF]
